# Immunesenescence: A Predisposing Risk Factor for the Development of COVID-19?

**DOI:** 10.3389/fimmu.2020.573662

**Published:** 2020-10-06

**Authors:** Jon Hazeldine, Janet M. Lord

**Affiliations:** ^1^Medical Research Council-Versus Arthritis Centre for Musculoskeletal Ageing Research, Institute of Inflammation and Ageing, University of Birmingham, Birmingham, United Kingdom; ^2^National Institute for Health Research Surgical Reconstruction and Microbiology Research Centre, Queen Elizabeth Hospital Birmingham, Birmingham, United Kingdom; ^3^National Institute for Health Research Birmingham Biomedical Research Centre, University Hospital Birmingham National Health Service Foundation Trust and University of Birmingham, Birmingham, United Kingdom

**Keywords:** aging, COVID-19, immunesenescence, immune dysfunction, inflammaging, SARS-Cov_2

## Abstract

Bearing a strong resemblance to the phenotypic and functional remodeling of the immune system that occurs during aging (termed immunesenescence), the immune response to severe acute respiratory syndrome coronavirus 2 (SARS-CoV-2), the causative agent of Coronavirus disease 2019 (COVID-19), is characterized by an expansion of inflammatory monocytes, functional exhaustion of lymphocytes, dysregulated myeloid responses and the presence of highly activated senescent T cells. Alongside advanced age, male gender and pre-existing co-morbidities [e.g., obesity and type 2 diabetes (T2D)] are emerging as significant risk factors for COVID-19. Interestingly, immunesenescence is more profound in males when compared to females, whilst accelerated aging of the immune system, termed premature immunesenescence, has been described in obese subjects and T2D patients. Thus, as three distinct demographic groups with an increased susceptibility to COVID-19 share a common immune profile, *could immunesenescence be a generic contributory factor in the development of severe COVID-19?* Here, by focussing on three key aspects of an immune response, namely pathogen recognition, elimination and resolution, we address this question by discussing how immunesenescence may weaken or exacerbate the immune response to SARS-CoV-2. We also highlight how aspects of immunesenescence could render potential COVID-19 treatments less effective in older adults and draw attention to certain therapeutic options, which by reversing or circumventing certain features of immunesenescence may prove to be beneficial for the treatment of groups at high risk of severe COVID-19.

## Introduction

Severe acute respiratory syndrome coronavirus 2 (SARS-CoV-2) is a novel highly-infectious betacoronavirus originally found in Wuhan, China in December 2019 (1). Transmitted by direct contact with infected individuals, contaminated surfaces or via respiratory droplets, SARS-CoV-2 is the causative agent of Coronavirus disease 2019 (COVID-19), which as of June 2020 had infected over 7 million people resulting in over 400,000 deaths (2). Whilst for the majority of individuals COVID-19 is a self-resolving mild to moderate respiratory tract infection, ~20% of infected patients develop severe respiratory complications (e.g., dyspnea and pneumonia), which, in extreme cases (~5%), progress to acute respiratory distress syndrome (ARDS), respiratory failure, organ damage, and death (3–6).

Epidemiological analyses of COVID-19 outbreaks have revealed the disease to be highly prevalent amongst older adults, with one study of 1,591 patients reporting 87% of cases were in adults aged 51 years and over (7, 8). Furthermore, older adults are more prone to developing severe COVID-19 and its associated poor outcomes (4, 6, 9–15). For example, 91 and 81% of COVID-19 related deaths have occurred in people aged 65 years and over in the UK and USA respectively, with the majority of deaths occurring in those aged 85 years and over (16, 17). Moreover, the recovery times of older adults who survive COVID-19 are more protracted, involving more serious clinical manifestations that often require hospitalization and prolonged therapy (10, 14, 18).

The scientific community has moved rapidly to gain an understanding of the immune response to SARS-CoV-2 and how it influences patient outcome. Summarized recently by Vabret et al. (19) the current literature details a hyper-inflammatory state in severe COVID-19 patients that is characterized by a sustained raised level of pro-inflammatory cytokines such as interleukin (IL)-6, expansion of inflammatory monocytes and T cells, dysregulated myeloid responses, functional exhaustion of lymphocytes and impaired innate immune function. This immunological profile bears a strong resemblance to the remodeling of the immune system that occurs during physiological aging. Termed immunesenescence, immune aging is associated with marked alterations in the composition, phenotype and functional responsiveness of the innate and adaptive arms of the immune system that compromises the older adults ability to combat infections allowing for pathogen dissemination in a vicious cycle that leads to further inflammation and ultimately tissue damage. Furthermore, aging is accompanied by a state of chronic low-grade systemic inflammation, termed *inflammaging*, meaning older patients start with a higher inflammation status prior to infection. Immunesenescence is viewed as a major contributory factor in the increased susceptibility of older adults to infection (20, 21) as well as their poor vaccination responses (22). In addition to older adults, males (3, 4, 6, 13, 23, 24) as well as patients with pre-existing co-morbidities such as diabetes (4, 13–15, 25) and obesity (11, 24, 26–29) are at an increased risk of severe COVID-19.

Immunologically, immunesenescence and *inflammaging* appear to be more profound in older males when compared to females (30, 31), whilst an accelerated aging phenotype, termed premature immunesenescence has been described in obese subjects and patients with type 2 diabetes (T2D) (32–34). Although 85–90% of T2D patients are overweight or obese, not all adults who are obese develop T2D and most studies suggest the prevalence is below 50% (35). For this reason, we have considered three distinct demographic groups with an increased susceptibility to COVID-19 that appear to share a common immune profile, posing the question *could immunesenescence be a generic contributory factor in the development of severe COVID-19?* Here, by focussing on three key aspects of an immune response, namely pathogen recognition, elimination and resolution, we will address this question by discussing how immunesenescence may weaken or exacerbate the immune response to SARS-CoV-2. We also highlight how aspects of immunesenescence could render potential COVID-19 treatments less effective in older adults and draw attention to certain therapeutic options, which by reversing or circumventing certain features of immunesenescence may prove to be beneficial for the treatment of groups at high risk of severe COVID-19.

## Pathogen Recognition

### Pathogen Recognition Receptor Expression and the Early Anti-viral Response

Comprised of four different families, namely the toll-like receptors (TLRs), retinoic acid-inducible gene (RIG)-I-like receptors (RLRs), nucleotide-binding oligomerization domain-like receptors (NLRs) and the C-type lectin receptors (CLRs), pathogen recognition receptors (PRRs) are evolutionary conserved germline-encoded receptors responsible for the early detection of invading pathogens. Located at the cell surface, in endosomes and in the cytosol, PRRs are expressed predominantly by cells of the innate immune system, in particular monocytes and dendritic cells (DCs). As a single-stranded RNA virus, detection and initiation of the immune response against SARS-CoV-2 will be mediated by the RNA-sensing endosomal PRRs TLR 3, 7 and 8, and the cytoplasmic-residing RLRs and NLRs.

Ligation of PRRs activates interferon regulatory factors (IRFs), a family of transcription factors that drive the production of type I (α/β) and type III (γ) interferons (IFNs) (36). By inhibiting viral replication, enhancing innate immune responses and modulating T cell expansion and memory formation (37), IFNs provide strong anti-viral effects. SARS-CoV-2 appears particularly sensitive to IFNs, with *in vitro* culture studies revealing viral replication in kidney epithelial cells and primary human intestinal epithelial cells is potently inhibited by type I and type III IFNs, respectively (38, 39). In one of the few studies to have investigated the IFN response to SARS-CoV-2 in patients (40, 41), Hadjadj et al. identified a distinct type I IFN signature in severe COVID-19 patients (40). Compared to individuals with mild to moderate disease, critically ill patients presented with marked downregulation of IFN-stimulated genes in whole blood leukocytes, significantly lower plasma levels of IFN-α2 and reduced IFN activity in serum (40).

Studies that have examined the effect of age on the expression of RNA-sensing PRRs have reported significantly reduced expression of TLRs 3, 7, and 8 in myeloid DCs (mDCs) or plasmacytoid DCs (pDCs) isolated from older adults (42, 43). Accompanying these changes in PRR expression is an age-related impairment in the generation of type I and III IFNs (44). pDCs or monocytes from older adults secrete significantly lower amounts of IFN α, β, or γ in response to specific ligation of TLRs 7/8 and RIG-I, with the reduction in IFN α and β synthesis post-RIG-I activation attributed to impaired activation of IRFs (43, 45–47). Furthermore, and of particular importance in the context of SARS-CoV-2, age-related impairments in type I IFN production have been described for monocytes and pDCs challenged with influenza A virus and West Nile virus (WNV) (42, 47–50), two RNA viruses that also cause significant morbidity and mortality in older adults (51–53). As prompt and efficient type I IFN responses are critical for preventing poor outcome following coronavirus infections (54, 55), an age-related impairment in IFN production may result in more robust virus replication and higher viral loads. On this note, it has been suggested that COVID-19 patients with type I IFN deficiency, a criterion we propose older adults would fulfill, may benefit from IFN α or β supplementation (40). In an open-label, randomized, phase 2 trial in COVID-19 patients, Hung and colleagues demonstrated that, when compared to anti-viral drug treatment alone, a combined therapy of anti-viral drugs and IFN-β significantly shortened the duration of viral shedding, time to symptom resolution and length of hospital stay in patients with mild to moderate disease (56). Whilst this therapeutic approach is worthy of consideration for geriatric COVID-19 patients, it should be noted that *in vitro* studies with monocytes from older adults have demonstrated reduced up-regulation of IFN-stimulated genes following influenza A virus challenge (47). Thus, increasing type I IFN levels in older adults via IFN supplementation may be offset by an age-related impairment in IFN responsiveness.

Ligation of PRRs also triggers the secretion of pro-inflammatory cytokines via the activation of nuclear factor kappa B (NF-κB) and mitogen activated protein kinase (MAPK) signaling pathways. Compared to those with mild-to-moderate disease, patients with severe COVID-19 infection present with significantly elevated circulating concentrations of a range of pro-inflammatory cytokines such as IL-6 and tumor necrosis factor-alpha (TNF-α) (57–61). Although not observed in all studies (43, 62), the majority of groups that have investigated cytokine production triggered by RNA-sensing PRRs have found this function is maintained with age (42, 43, 46, 47, 62). For example, in response to stimulation with TLR3, TLR 7/8 and RIG-I specific ligands, as well as influenza A virus, mDCs or monocytes isolated from young and older adults generate comparable levels of TNF-α, IL-6 and/or IL-12 (42, 43, 46, 47, 62). In the context of COVID-19, these data imply that the pro-inflammatory cytokine response to SARS-CoV-2 elicited by monocytes and mDCs would be similar across different age groups. However, this may not be the case for patients with pre-existing co-morbidities. For instance, compared to normal-weight controls, monocytes isolated from obese subjects generate significantly greater amounts of TNF-α and CCL5 following stimulation with viral ssRNA (63), whilst in monocytes from T2D patients, basal expression of components of the TLR signaling pathway such as the adaptor proteins MyD88 and TRIF as well as the p65 subunit of NF-κB are significantly increased (64). Thus, we propose that this remodeling of innate immune cells in obese and T2D patients would lead to a more robust pro-inflammatory response to SARS-CoV-2 when compared to that of healthy age-matched controls, culminating in greater systemic inflammation and more severe disease.

Generated via the activation of the NLRP3 inflammasome, a multi-subunit complex comprising of the NLR protein NLRP3, the adaptor protein ASC and caspase-1, IL-1β promotes anti-microbial resistance via the modulation of innate and adaptive immune responses (65). However, if dysregulated, production of this pro-inflammatory cytokine can promote lung injury and severe pulmonary fibrosis (66, 67). Coinciding with elevated plasma levels of IL-1β (3), single cell transcriptomic analysis of peripheral blood mononuclear cells (PBMCs) has shown a greater abundance of classical CD14^++^ CD16^−^ IL1β^+^ monocytes in COVID-19 patients when compared to healthy controls (HCs) (68), whilst analysis of RNA extracted from whole blood found increased IL-1β gene expression preceded a decline in respiratory function (69). In terms of patient groups at high risk of severe COVID-19, significantly increased NLRP3 expression and ssRNA-induced IL-1β generation has been reported for monocytes and monocyte-derived macrophages isolated from T2D patients and obese subjects, respectively (63, 70, 71), suggesting potential exaggeration of inflammasome-mediated immune responses to SARS-CoV-2 in these cohorts. Conversely, aging appears to be associated with impaired activation of the inflammasome. Investigated primarily in animal models, significantly reduced inducible expression of NLRP3, ASC and/or caspase-1 has been described in lung homogenates, macrophages and/or DCs from aged mice, with these changes in expression resulting in decreased synthesis of IL-1β upon stimulation (72–74). Highlighting the importance of the inflammasome in host protection, models of influenza infection and secondary *Streptococcus pneumoniae* infection have shown the age-associated decrease in NLRP3 inflammasome expression and activity results in impaired cell infiltration to sites of infection, increased pathogenic load in the lung and higher rates of morbidity and mortality (72, 74). In terms of human aging and its impact on the inflammasome, no change (47) or a significant reduction (45) in IL-1β production by monocytes challenged with influenza A virus or TLR 7/8 ligands, respectively has been reported. Given the importance of the inflammasome in host defense against viral infections (75, 76), we suggest that the older COVID-19 patient with no pre-existing co-morbidities would elicit an impaired inflammasome-mediated immune response to SARS-CoV-2 that would increase their susceptibility to severe disease.

## Pathogen Elimination

### Neutrophils

Currently, few studies have reported upon the neutrophil response to SARS-CoV-2. These studies have shown neutrophilia (3, 4), an elevated neutrophil-to-lymphocyte ratio (60, 61, 77–79) and neutrophil infiltration in the lungs (80, 81) to be features of severe COVID-19 and poor patient outcomes. In the only laboratory-based study, Zuo et al., using cell-free DNA (cfDNA), myeloperoxidase-DNA complexes and citrullinated histone H3 as surrogate markers of *in vivo* neutrophil extracellular trap (NET) formation, reported elevated levels of all three markers in serum samples obtained from hospitalized COVID-19 patients when compared to HCs (82). Significantly higher cfDNA and myeloperoxidase-DNA complexes were recorded in those who required mechanical ventilation, suggesting a potential relationship between enhanced NET formation and disease severity (82). Previously linked to the pathogenesis of acute lung injury and the onset of ARDS in critically-ill patients (83–86), the authors suggested robust NET formation may propagate the inflammatory storm that appears to precede the onset of severe COVID-19 (3, 82, 87).

In the context of immunesenescence, both murine and human-based studies have reported a significant age-related reduction in NET formation (88–90). Thus, in contrast to younger adults and those with inflammatory co-morbidities (91–93), we would speculate that in older adults with no pre-existing health conditions, any elevation in circulating NET components post SARS-CoV-2 infection would not be a direct consequence of enhanced NET formation. Rather, we suggest that reduced clearance may be responsible. Once dismantled by the endonuclease deoxyribonuclease (DNase)-1, NETs are engulfed by macrophages and degraded in lysosomes, a process facilitated by the opsonisation of NET fragments by the complement protein C1q (94). Whilst no study to our knowledge has investigated the effect of age on DNase-1 activity, there are reports that aging is associated with reduced endocytic and phagocytic activity of macrophages (95–97) as well as reduced lysosomal activity (98). When viewed alongside data from critically-ill patients, in whom DNase activity and uptake of NETs by alveolar macrophages (AM) is significantly reduced (99, 100), then older adults with severe COVID-19 are a group that would be predicted to present with a high systemic NET load, a scenario, which in a cohort of patients with severe influenza A infection was associated with the development of multiple organ dysfunction syndrome (101).

NET production, whether assessed by a measurement of circulating markers (e.g., MPO-DNA complexes) or *ex vivo* generation, is significantly increased in obese subjects and individuals with T2D (91–93), patient groups that are not only at high risk of developing severe COVID-19 (102, 103) but who experienced poor outcomes in the 2009 H1N1 influenza A virus pandemic (104). Whilst multiple factors will underlie the susceptibility of obese and T2D subjects to severe COVID-19, it is intriguing to speculate that remodeling of the innate immune response, in this case a heightened sensitivity for NET generation, could be one such factor, particularly given the cytotoxic and pro-thrombotic nature of NETs (105, 106).

NETs may represent a potential therapeutic target for the prevention of poor outcomes such as ARDS in COVID-19. In a recent article, Barnes et al. discussed the therapeutic options that are available to manipulate NET formation and how some of these approaches are already being tested in clinical trials in COVID-19 patients (80). Improvements in clinical indices were reported in a cohort of severe COVID-19 patients that were co-treated with anti-viral agents and dipyridamole, an adenosine-receptor agonist that inhibits NET formation *in vitro* (107, 108). However, whether the observed benefits were related to the modulation of NET production was not addressed (108). Nevertheless, the success that enhancing NET degradation has had in terms of improving clinical markers in patients with virus-associated bronchiolitis (109, 110) and reducing both lung injury and mortality rates in murine models of pneumonia (84), should encourage researchers and clinicians to pursue NETs as therapeutic strategies. This is particularly pertinent to older adults, where administration of therapeutic doses of DNase would completely eradicate NETs (94), thereby bypassing the need for macrophage clearance, which is a process that is likely to be impaired with age.

Associated with lymphocytic and neutrophilic infiltrate, post-mortem histological examination of lung tissue has shown severe COVID-19 results in extensive diffuse alveolar damage (81). In response to a panel of inflammatory mediators, which included IL-8, C5a, leukotriene B4 and sputum, we have shown aging is associated with impaired migratory accuracy of neutrophils (111). This defect, which was detected in individuals aged ≥60 years, was accompanied by enhanced degranulation and neutrophil proteinase activity, leading us to propose that aging is associated with an increase in neutrophil-mediated bystander tissue damage (111). Interestingly, a similar situation may be observed in T2D patients, whose neutrophils also exhibit impaired migration *in vitro* (112, 113). Furthermore, compared to HCs, circulating levels of the protease inhibitor alpha-1 antitrypsin are significantly lower in T2D subjects (114). Thus, in the context of SARS-CoV-2, we suggest that the meandering neutrophils of both older adults and T2D patients would, via excessive proteinase release, promote more widespread tissue damage and increased systemic inflammation.

### Monocytes and Macrophages

Accompanied by an emergence into circulation of large atypical vacuolated monocytes (115), SARS-CoV-2 infection is associated with alterations in the composition of the peripheral monocyte pool. For example, whereas frequencies of CD14^++^ CD16^−^ classical monocytes have been reported to be significantly reduced in COVID-19 patients when compared to HCs (115), the proportions of intermediate (CD14^++^16^+^) and non-classical (CD14^+^16^++^) monocytes are significantly increased (115, 116), with analysis also revealing the percentage of intermediate CD14^++^16^+^ monocytes to be significantly higher in patients requiring intensive care unit (ICU) treatment when compared to those with milder disease (116).

Moreover, single cell analysis of PBMCs has reported the presence of a monocyte subset unique to severe COVID-19 patients that is enriched in genes encoding a range of cytokine storm related cytokines such as IL-1β, IL-6, and TNF-α (117). Phenotypically, mirroring the immunological changes that occur during sepsis, monocytes from COVID-19 patients exhibit significantly reduced surface expression of the antigen presenting molecule HLA-DR (118). *Ex vivo* examination of intracellular cytokine levels has revealed an increased frequency of GM-CSF^+^ and IL-6^+^ monocytes in both ICU and non-ICU COVID-19 patients, with the percentage of IL-6^+^ monocytes correlating with disease severity (116). Similarly, a greater proportion of CD14^++^ CD16^−^ IL1β^+^ monocytes were detected in COVID-19 patients by RNA sequencing, which found expression in CD14^++^ monocytes of pro and anti-inflammatory genes were up and down-regulated, respectively when compared to HCs (68). Whilst more studies are required, emerging data implies a role for IL-6 in driving the SARS-CoV-2-mediated remodeling of the monocyte pool (117, 118), with one group demonstrating a significant reduction in the expression of genes involved in “leukocyte chemotaxis” and the “acute inflammatory response” in monocytes obtained from COVID-19 patients following treatment with the IL-6 receptor monoclonal antibody Tocilizumab (117).

Physiological aging and obesity are associated with remodeling of the circulating monocyte pool, with older adults and obese subjects exhibiting elevated frequencies of intermediate and non-classical monocytes when compared to younger adults and lean subjects, respectively (119–126). Interestingly, Ong et al. have recently assigned a senescent-like pro-inflammatory phenotype to both non-classical and intermediate monocytes (123). Associated with high expression of the phosphorylated p65 subunit of NK-κβ, both monocyte subsets secreted, in the absence of *ex vivo* stimulation, an array of pro-inflammatory cytokines and chemokines, which included TNF-α, IL-6, and CCL4 (123). Importantly, this basal increase in monocyte activity was associated with significantly elevated plasma levels of IL-6 and TNF-α (123). Thus, in the absence of infection, obese and older adults exhibit a state of heightened peripheral inflammation upon which the abovementioned SARS-CoV-2-mediated changes in monocyte biology would be super-imposed. When combined with the maintained (42, 43, 46, 47, 62) or increased (63) generation of pro-inflammatory cytokines by RNA-stimulated monocytes of older adults and obese subjects respectively, we speculate that this high level of basal inflammation would predispose these groups to hyper-inflammation that would hasten the onset of severe COVID-19.

Single cell RNA sequencing (scRNA-seq) of bronchoalveolar lavage fluid (BALF) has revealed the composition of macrophages within the lungs of COVID-19 patients differs based on disease severity. Categorizing macrophages as monocyte-derived, pro-fibrotic or alveolar, Liao et al. found BALF obtained from patients with severe disease was dominated by monocyte-derived and pro-fibrotic macrophages, with the former subset expressing a strong pro-inflammatory gene signature (127). Offering potential insights into the secondary complications that may develop in severe COVID-19 patients as a consequence of this remodeling of lung-resident macrophages, two studies have implicated monocyte-derived AMs in the development of post-injury lung fibrosis and viral-induced pneumonia (128, 129). In the context of immunesenescence, it has been proposed that as a consequence of life-long exposure to environmental challenges, monocyte recruitment to the lung increases with age, such that over time, monocyte-derived macrophages become the predominant subset within the lungs (130). If correct, then a more robust pulmonary inflammatory response to SARS-CoV-2 in older adults may increase their susceptibility to developing severe COVID-19.

### Natural Killer Cells

Natural killer (NK) cells are innate immune cells that play a major role in the early recognition and elimination of virally-infected cells. In a murine model of severe SARS-CoV-1 pulmonary infection, Glass et al. demonstrated viral clearance in the absence of NK cells (131), a finding that suggests these innate lymphocytes are not required for host protection against coronaviruses. However, the significant number of studies that have demonstrated marked alterations in the composition and function of the circulating NK cell pool of COVID-19 patients (19) makes a discussion of the NK cell response to SARS-CoV-2, particularly in the context of immunesenescence, necessary.

COVID-19 patients with mild-to-moderate disease present with significantly reduced circulating numbers of total NK cells, driven by a reduction in both CD56^DIM^16^+^ and CD56^BRIGHT^ NK cell subsets (40, 41, 79, 118, 132–134). Accompanying these numerical changes are significant alterations in NK cell phenotype, with scRNA-seq and flow cytometric analyses revealing the peripheral NK pool of COVID-19 patients is dominated by immature, highly activated and functionally compromised cells (40, 41, 134). Focussing on the latter, increased frequencies of NK cells expressing the inhibitory receptors TIM3 and NKG2A have been detected in patients with mild/moderate and severe COVID-19 (40, 134) with the increase in NKG2A expression potentially reflecting the stimulation of NK cells by pro-inflammatory cytokines (135). Upon recognition of its ligand HLA-E, signaling through NKG2A inhibits NK cell cytotoxicity (NKCC) (136, 137). Thus, one would predict that NK cells isolated from COVID-19 patients would exhibit reduced functional responses. Indeed, albeit to a non-viral stimulus, Zheng and colleagues found the frequencies of CD107a^+^, IFNγ^+^, TNFα^+^, and IL-2^+^ NK cells in PBMC samples acquired from COVID-19 patients were significantly lower following PMA and ionomycin challenge when compared to HCs (134). As blood samples were acquired at the time of hospital admission, these results imply an immediate breakdown of NK-mediated anti-viral immunity (134). Interestingly, when patients were reanalysed following anti-viral therapy, a marked reduction in the percentage of NKG2A^+^ NK cells was noted, leading to the suggestion that downregulation of NKG2A may correlate with disease control (134).

A prominent feature of NK cell immunesenescence is reduced NKCC, a defect we have previously attributed to impaired polarization of the pore forming protein perforin to the immunological synapse (138). Accompanying this decline in lytic activity is an age-related reduction in cytokine and chemokine production (139–141). NK cell function is regulated by the balance of signals transmitted through surface expressed activatory and inhibitory receptors (142). As discussed by others (19), it is currently unknown as to which ligands for activatory receptors are expressed on the surface of SARS CoV-2 infected cells. Possible candidates are stress-inducible ligands, which are recognized by the activatory receptors NKG2D, NKp30, and NKp46. Whilst age has no effect upon the expression of NKG2D (138, 143), a number of studies have described an age-associated decline in the frequency of NKp30^+^ and NKp46^+^ NK cells (138, 144, 145). Thus, in the older adult with severe COVID-19, superimposed on a baseline reduction in NKCC and activatory receptor expression would be a SARS-CoV-2 driven induction of functional exhaustion via the up-regulation of NKG2A (134). Moreover, with *in vitro* studies having shown that exposure to IL-6 and TNF-α, two cytokines whose circulating levels are elevated in COVID-19 patients (3, 77, 146) impairs NKCC and reduces perforin, NKp30 and NKp46 expression (147–149), then the SARS-CoV-2-induced cytokine storm would exacerbate the abovementioned functional and phenotypical features of NK cell immunesenescence, which would be predicted to further reduce NK cell anti-viral activity.

Recent studies in the field of cancer immunotherapy have shown that manipulation of NKG2A signaling can restore NKCC and promote anti-tumor immunity (136, 150, 151). Based on its success, Yaqinuddin and colleagues have proposed mirroring this therapeutic approach for the treatment of COVID-19 patients, where administration of the humanized anti-NKG2A antibody Monalizumab would rejuvenate the anti-viral immune response of COVID-19 patients by counteracting the NKG2A-driven inhibition of NKCC (152). However, for older adults with severe COVID-19, any therapeutic value of this approach may be offset by the age-related impairments in perforin polarization, NKCC and the reduced expression of NK cell activating receptors.

### T Cell Responses

Lymphopenia is a common hematological observation in patients infected with SARS-CoV-2. CD3^+^, CD4^+^, and CD8^+^ T cell counts are significantly lower in patients with severe COVID-19 when compared to those with mild disease (57, 60, 133, 134, 153), with numbers increasing significantly in subjects who respond clinically to anti-viral treatment (133). Elevated circulating concentrations of pro-inflammatory cytokines (133, 154), induction of apoptosis (40) and pulmonary infiltration (5, 127) are some of the mechanisms that have been proposed to underlie SARS-CoV-2-induced lymphopenia. Indicative of *in vivo* activation, increased proportions of CD4^+^ and CD8^+^ T cells expressing CD69, CD38, CD44, or HLA-DR have been reported in COVID-19 patients (5, 116, 155–158) as has the presence of pathogenic GM-CSF^+^/IL-6^+^ and GM-CSF^+^/IFN^+^ CD4^+^ T cells, with those experiencing severe disease presenting with significantly increased frequencies when compared to those with mild COVID-19 (116). Pointing toward a state of functional exhaustion or senescence, markedly higher percentages of CD4^+^ or CD8^+^ T cells expressing a variety of molecules such as NKG2A, PD-1, TIGIT, TIM-3 and CD57 have been detected in SARS-CoV-2-infected patients (116, 134, 154, 159), with their presence coinciding with significantly reduced intracellular cytokine generation upon *ex vivo* stimulation (134, 158).

Characterized by the gradual replacement of functional epithelial cells with fat and fibrous tissue (160), thymic involution is a defining feature of T cell immunesenescence, which results in a decline in the production of naïve T lymphocytes (161). This reduction in thymic output is offset by the homeostatic proliferation of pre-existing naïve and memory T cells, a scenario that results in a contraction in the diversity of the circulating T cell receptor (TCR) repertoire of older adults (162). As well as aging, obesity is associated with reduced thymic function. Yang and co-workers found the generation of naïve T cells was significantly lower in obese younger adults when compared to age-matched lean controls (163). As a broad TCR repertoire is crucial for the detection of novel pathogens, the reduced diversity within the T cell pool of older adults and obese subjects may contribute to their increased susceptibility to SARS-CoV-2 infection and put them at risk of eliciting a blunted immune response to any future COVID-19 vaccine.

Owing to impaired metabolism, shortened telomeres and aberrant intracellular signaling (33, 164, 165), reduced proliferation, cytokine production, cytotoxicity and migration are examples of some of the functional impairments that have been reported for T cells isolated from older adults and those with inflammatory co-morbidities (33, 166, 167). The peripheral T cell pools of these adults are enriched with functionally exhausted (TIGIT^+^, PD-1^+^), highly activated (TIGIT^+^ HLA-DR^+^ CD38^+^), senescent (CD28^−^57^+^, CCR7^−^45RA^+^) and terminally differentiated (CD27^−^28^−^) CD4^+^ or CD8^+^ T cells (33, 168–170). The most profound changes are witnessed within the CD8^+^ T cell subset, with the accumulation of CD8^+^28^−^ T cells of particular significance (171). Saurwein-Teissl et al. found an expansion of CD8^+^28^−^ T cells was associated with reduced antibody responses in older adults following influenza vaccination (172). The efficiency of T cell responses are also hampered by age-associated alterations in the expression of co-stimulatory molecules on the surface of antigen presenting cells. Relevant to SARS-CoV-2, monocytes isolated from older adults have been shown to exhibit reduced expression of CD80 and CD86 following ligation of the RNA-sensing PRRs TLR7/8 (173). In subsequent vaccination studies, it was shown that expression of these co-stimulatory molecules was positively associated with antibody responses (173).

Based on scRNA-seq data that has shown the presence of highly expanded and functionally-competent CD8^+^ T cells in the BALF of mild COVID-19 patients, it has been suggested that a robust adaptive immune response is critical to controlling SARS-CoV-2 infection (127). If correct, then combined with the aforementioned remodeled T cell pool of older adults and individuals with inflammatory co-morbidities, the SARS-CoV-2 driven induction of lymphocyte exhaustion (116, 134, 154, 159) would hamper both the initiation and maintenance of such a response. Furthermore, due to the reduced vaccine efficacy that occurs as a consequence of both innate and adaptive immune dysfunction, alternative therapeutic strategies such as administration of the immunomodulatory drugs metformin and pioglitazone, have been proposed to protect these high risk groups against severe COVID-19 (174).

### B Cells

Marked alterations have been described in the composition of the circulating B cell pool of SARS-CoV-2 infected patients. Relative to HC's, significantly reduced frequencies of naïve IgM^+^CD27^−^, memory CD21^+^27^+^ and CD5^+^ B cells have been reported (175, 176), and are accompanied by a concurrent elevation in the proportion of CD38^+^27^+^ plasmablasts (175–177). When analyzed by disease severity, significant alterations in plasmablast and memory CD21^+^27^+^ B cell frequencies were observed only in patients with severe COVID-19 disease, with the proportions of both subsets returning to levels comparable to those of HCs upon recovery (175, 176). Demonstrating a rapid and robust B cell response to SARS-CoV-2 infection, elevated circulating levels of virus specific IgM, IgG, and IgA antibodies have been detected, with this seroconversion evident within 7–14 days post-symptom onset (19, 68, 175, 176, 178). Interestingly, in a small pilot study of five critically-ill COVID-19 patients, transfusion of convalescent plasma containing neutralizing SARS-CoV-2 specific antibodies was shown to improve clinical status (179). In terms of the longevity of the antibody response, SARS-CoV-2 specific IgG antibodies have been detected in serum samples acquired from COVID-19 patients 7 weeks post-infection (180). However, due to the infancy of the current pandemic, it is currently unknown as to whether this initial antibody response and generation of memory B cells will protect against re-infection. That said, data from previous coronavirus outbreaks, in which a progressive decline in both SARS-CoV-1 specific IgG memory B cells and IgG antibodies were reported (181, 182), suggests that SARS-CoV-2 antibody responses will wane over time.

Attributed to a range of factors such as changes in the bone marrow microenvironment and skewing of haematopoietic stem cell differentiation toward the myeloid lineage, murine-based studies have shown aging is associated with a reduction in mature B cell production (183). In line with this observation, human aging is accompanied by a reduction in the size of the peripheral B cell pool, with both the frequency and absolute numbers of CD19^+^ B cells significantly lower in older adults (183–185). However, whether human aging is associated with changes in the composition of the peripheral B cell pool is unclear. For example, whilst some groups have reported an age-related increase in the percentage or number of circulating CD27^+^ memory B cells (185), others have demonstrated an age-associated decline in this subset (183, 184). Similarly, the frequency of IgM memory B cells have been reported to be either decreased (186) or unchanged with age (184).

Results of human and animal-based studies have revealed that aging is associated with reduced B cell proliferation and differentiation into plasma cells, which secrete antibodies that are weaker and of lower affinity when compared to those produced by plasma cells of younger subjects (186–189). Critical steps in a humoral immune response are class switch recombination (CSR) and somatic hypermutation (SHM). Taking place in germinal centers, these two processes are responsible for the generation of isotype-switched high-affinity antibodies. Essential for both CSR and SHM is activation-induced cytidine deaminase (AID), a DNA-editing enzyme, whose expression is regulated by the transcription factor E47. Culminating in defective class switching, the expression of both AID and E47 has been shown to be significantly lower in B cells from aged mice and humans (184, 190, 191). Alongside these intrinsic defects, B-cell extrinsic factors also contribute to the age-related impairment in humoral immunity. For example, attributed to reduced surface expression of Fc receptors, follicular dendritic cells of aged mice exhibit reduced antigen trapping and presentation (192), whilst the age-related decline in CD40L expression on the surface of activated CD4^+^ T cells would reduce the delivery of co-stimulatory signals to antigen-expressing B cells (193).

B cell immunesenescence is considered a major underlying factor in the reduced efficacy of vaccination in older adults. Characterized by decreased antibody concentrations, delayed peak antibody titres and lower seroprotection (194–196), the humoral response to a range of vaccinations such as influenza (197) and Hepatitis A (196) is significantly reduced in older adults. Furthermore, accompanying this impairment in initial antibody responses is an age-associated decline in antibody persistence, with one study reporting non-protective antibody titres to be present in older adults 6–10 years following vaccination with tetanus toxoid (198). In the context of COVID-19, these studies highlight the need for research groups involved in designing a SARS-CoV-2 vaccine to consider the impact that age will have on its efficacy, and whether one vaccine will confer protection amongst all groups of society. With this in mind, it may be that a vaccination strategy specific for older adults is required. This could involve the co-administration of an adjuvant or delivery of a booster vaccine, two strategies that have previously proven successful in augmenting antibody titres and conferring seroprotection in aged rhesus monkeys and humans (199, 200).

### Inflammaging

Physiological aging is accompanied by a sub-clinical chronic low-grade state of systemic inflammation, *inflammaging*. This phenomenon is characterized by elevated serum levels of acute phase proteins (e.g., C-reactive protein) and pro-inflammatory cytokines (e.g., TNF-α, IL-6, and IL-8) (201). Previous papers that have discussed COVID-19 in the context of aging and immunesenescence have speculated that *inflammaging* would predispose the older adult to severe infection by fuelling an exaggerated pro-inflammatory response to SARS-CoV-2 (202, 203). However, based on emerging data that suggests excessive pro-inflammatory responses in older adults negatively regulates their immune responses (204, 205), we propose the following alternative hypothesis: *inflammaging predisposes older adults to severe COVID-19 by suppressing the immune response to SARS-CoV-2*. Whilst *in vitro and in vivo* studies have demonstrated that exposure to pro-inflammatory cytokines can modulate the phenotype and/or function of innate and adaptive immune cells (147–149, 206), it is the work of Akbar et al. that have specifically linked hyper-inflammation to impaired antigen specific immune responses during aging. Using a human experimental system that investigates antigen-specific immunity *in vivo*, the group has consistently demonstrated an age-related impairment in the delayed type hypersensitivity (DTH) response to varicella zoster virus (VSV) antigen (204, 205, 207). Attributed to aberrant activation of P38 MAPK signaling, the decreased VZV antigen responsiveness of older adults is associated with an accumulation of CCR2^+^ monocytes that inhibit T cell proliferation via the production of prostaglandin E2 (PGE_2_) (204, 205).

In terms of COVID-19, it is interesting that the aging lung is characterized by a state of heightened basal inflammation, with levels of IL-6, amongst other cytokines, significantly higher in the BALF of healthy older adults when compared to their younger counterparts (208–210). It has been suggested that a life-long accumulation of senescent cells may be responsible for this age-associated increase in pulmonary inflammation (210). Whilst data from murine models support this assumption (211), it is currently unknown in humans as to whether aging is associated with an increased senescent cell burden in the lungs. However, it is interesting that metatranscriptomic sequencing of BALF from COVID-19 patients aged 40-61 years detected an up-regulation of CCL2 (212), a chemokine produced in large amounts by senescent cells (213, 214). Moreover, CCL2 is the chemoattractant for CD14^++^ CCR2^+^ classical and intermediate monocytes, which in the abovementioned VZV models were more abundant at sites of antigenic challenge in older adults and negatively regulated the adaptive immune response (204). Thus, in response to SARS-CoV-2, the pulmonary immune response of older adults may share features reminiscent of the impaired cutaneous immune response described in DTH models, in that, via the CCL2-mediated recruitment of PGE_2_-secreting monocytes, a hyper-inflammatory response would impede T cell function.

Residing in a state of permanent cell cycle arrest, yet remaining metabolically active, senescent cells are a rich source of pro-inflammatory cytokines, chemokines, growth factors and proteases (215). Due to this inflammatory profile, termed the senescent associated secretory phenotype (SASP), and their presence in various tissues of older adults and T2D patients (216–218), senescent cell accumulation is considered to be one factor underlying the heightened systemic inflammatory status of these individuals. Recently, it was demonstrated that certain viruses such as influenza virus exhibit enhanced replication efficiency in senescent cells (219). In terms of coronavirus, entry of SARS-CoV-1 into host cells has been shown to be dependent upon surface expression of vimentin, a filament protein that interacts directly with the spike protein of SARS-CoV-1 (220). Since vimentin was recently found to be expressed on the surface of senescent lung fibroblasts (221), and the fact that SARS-CoV-1 and SARS-CoV-2 utilize the same mechanism of attachment to host cells, a number of groups have proposed increased SARS-CoV-2 replication would occur in individuals with a high senescent cell burden (222–224). Thus, an increased presence of senescent cells may predispose to the development of severe COVID-19 via two mechanisms: (1) reduced immune cell clearance by contributing to the aforementioned inflammation-induced suppression of innate and adaptive immunity (204, 205, 225) and (3) increasing viral load by acting as a site of enhanced SARS-CoV-2 replication. Interestingly, a number of clinical trials assessing the therapeutic benefit of drugs that directly eliminate senescent cells or suppress their SASP are already underway in patients with COVID-19 (223, 226). Results of such studies will help researchers address whether a high senescent cell burden is indeed a risk factor for the development of severe COVID-19.

Belonging to one of two distinct subsets, namely monocytic or granulocytic, myeloid-derived suppressor cells (MDSC's) are a heterogeneous collection of immature cells. Via a range of mechanisms, which include the generation of ROS and nitric oxide, arginine metabolism, induction of T regulatory cells and the production of anti-inflammatory cytokines, MDSC's are potent immune suppressors, inhibiting the proliferation and activation of innate (NK cells, DC's and macrophages) and adaptive (T and B cells) immune cells (227, 228). Whilst the presence of MDSC's during acute inflammatory responses is seen as beneficial (due to their involvement in the resolution of inflammation), in the setting of chronic inflammation, where MDSC's persist, their suppressive activity is considered detrimental to the host (229). Thus, the elevated frequency of MDSC's reported in older adults, obese subjects and T2D patients (230–232) has been proposed as a potential mechanistic explanation for the increased susceptibility to infection and poor vaccination responses elicited by these individuals (233, 234). Given that such inflammatory mediators as PGE_2_, IL-6, TNF-α, and GM-CSF promote the expansion and activation of MDSC's (227), the hyperactive immune response and cytokine storm described in SARS-CoV-2-infected patients has resulted in a handful of studies investigating whether MDSC's may contribute to the pathogenesis of COVID-19.

Relative to HC's, significantly elevated frequencies of MDSC's (235) and granulocytic-MDSC's (G-MDSCs) (236) have been detected in peripheral blood samples obtained from mild and severe COVID-19 patients. Suggestive of driving reduced anti-viral immune responses, significant negative associations were reported between MDSC frequency and the percentage of perforin^+^ CD3^+^T cells and perforin^+^ NK cells (235), whilst in *ex vivo* cultures, depletion of G-MDSC's from PBMC samples of severe COVID-19 patients restored the proliferative capacity and cytokine production of T cells (236). In terms of disease severity, MDSC frequency has been reported to be significantly higher in patients with severe COVID-19 when compared to subjects with mild disease (236), whilst single cell transcriptomics has revealed the presence of immature CD14^+^MPO^+^Ki67^+^HLA-DR^lo^ suppressive monocytes and immature ARG1^+^CD101^+^S100A8/A9^+^ neutrophils only in patients with severe disease (237). Furthermore, there is evidence to suggest that MDSC's persist in severe patients, with one study reporting G-MDSC's comprised >30% of total PBMC's in samples acquired from 3 severe COVID-19 patients at day 18 post-hospital admission (236). Thus, it has been hypothesized that a SARS-CoV-2-induced expansion of MDSC's may promote immune paralysis and that current therapeutic approaches targeting the cytokine storm may have the additional benefit of augmenting anti-viral immune responses by reducing MDSC proliferation and activation (235).

### Age-Associated Changes in Pulmonary Immune Responses

Thus, far, our discussion of how immunesenescence may predispose to severe COVID-19 has focussed on the changes that occur in the composition, phenotype and/or function of circulating immune cells. As a respiratory tract infection, it is important to discuss the pulmonary immune response.

As the resident immune cell of the lungs, studies that have examined the effect of age on the pulmonary immune response have focussed predominantly upon the AM. Gene profiling of resting AMs has shown aging induces wide-spread transcriptional changes in aged mice (97), with up-regulation of inflammatory pathways related to oxidative burst and IL-8 supporting the notion that aging is associated with heightened basal inflammation within the lung (97). Intertwined with this pulmonary inflammaging is reduced AM function (95, 97, 238), with the work of Hinojosa et al. suggesting the elevation in basal inflammation is linked to impaired cytokine production via an up-regulation in AMs of A20, a negative regulator of NK-κβ and MAPKs (239). As both these signaling elements function downstream of the RNA sensing PRRs TLR7/8 and RIG-1, pro-inflammatory cytokine production by AMs following SARS-CoV-2 stimulation may be reduced with age. This theory is supported by the significantly reduced production of IL-6 by AMs from aged mice following *ex vivo* stimulation with the TLR7/8 agonist R848, an impairment that was reported alongside a down-regulation of TLR8 gene expression and a reduced induction of genes related to IL-6 signaling in lung tissue from aged mice following viral infection (240). Other age-related defects reported in the pulmonary immune response include reduced NKCC (241), impaired migration of pulmonary DCs to draining lymph nodes (DLNs) (242), diminished virus-specific CD8^+^ T cell responses (242–245) and delayed immune cell infiltration (74, 245). Results of adoptive transfer experiments point toward an immune suppressive environment rather than cell-intrinsic defects as the cause of some of the abovementioned functional impairments, with one study attributing the age-associated impairment in pulmonary DC and T cell responses to elevated levels of the immune suppressive eicosanoid PGD_2_ in the lungs of aged mice (97, 242).

Insights into how aging may specifically affect the pulmonary immune response to SARS-CoV-2 are offered by the results of murine and non-human primate models of SARS-CoV-1 infection (242, 246–251). Replicating the situation in humans, disease severity and lethality in these models are higher in aged animals when compared to their younger counterparts (242, 247–249), and interestingly, the immune dysregulation that occurs in aged mice infected with SARS-CoV-1 is greater than that detected during influenza A virus infection (242). Features of the pulmonary immune response of aged animals to SARS-CoV-1 include: reduced DC migration to DLNs (242), impaired CD8^+^ viral-specific T cell responses (242), decreased macrophage and DC activation (247), reduced T cell proliferation (247) and enhanced pro-inflammatory cytokine responses (246, 248, 249). Those studies that have reported an age-related increase in viral-induced inflammation have shown this exaggerated response is associated with significant lung damage, leading to the suggestion that a pathological immune response may contribute to the increased morbidity and mortality rates in older adults following coronavirus infection (246). Using two distinct approaches, namely antagonism of PGD_2_ signaling (242) or prophylactic treatment with the TLR3 agonist poly IC (251), it is possible to enhance the pulmonary immune response of older animals to SARS-CoV-1 and increase host survival (242, 251). Demonstrating reversal of immunesenescence, these therapeutic strategies have been proposed as a potential means of improving clinical outcome in older adults at high risk of severe respiratory infections (242, 251).

## Resolution of Inflammation

A co-ordinated multi-step program that involves the clearance of apoptosed neutrophils by macrophages (efferocytosis) and the generation of specialized pro-resolving lipid mediators (SPMs), the resolution of inflammatory responses is an active process that protects against unwarranted tissue damage (252). Whilst we await data relating specifically to features of the resolution phase in SARS-CoV-2-infected patients, a series of murine and human-based studies have shown aging (97, 225, 253), obesity (254, 255) and T2D (254, 256) are all associated with delayed resolution of inflammatory responses.

Attributed to a p38 MAPK driven reduction in the expression of T-cell immunoglobulin mucin protein 4 (TIM-4), a receptor expressed by macrophages that recognizes phosphatidylserine on the surface of apoptosed neutrophils, De Maeyer and colleagues recently demonstrated an age-associated impairment in efferocytosis (225). In a human dermal model of acute sterile inflammation, this defect in efferocytosis resulted in the accumulation of annexin V^+^ neutrophils and delayed resolution (225). Mirroring these observations, reduced clearance of apoptosed cells by macrophages has been reported in the experimental settings of obesity (97, 254, 257) and diabetes (254, 256, 258), with decreased PI3-K signaling (257) and elevated PGE_2_ levels in inflammatory exudate (254) identified as potential underlying causes. In addition to defective efferocytosis, reduced concentrations of SPMs have been measured at sites of acute inflammation in murine models of aging (253) and diabetes (256). Augmenting SPM levels via exogenous administration shortened resolution time *in vivo*, with this improvement linked to increased efferocytosis and the reprogramming of monocytes to a pro-resolving phenotype (253). Based on these data, we propose that, via their delayed induction of resolution programs, groups at high risk of COVID-19 would experience prolonged inflammatory responses following SARS-CoV-2 infection. By exacerbating their pre-existing heightened pro-inflammatory status, this impairment in resolution would promote further immune dysregulation and bystander tissue damage that would result in delayed viral clearance and an extended time to recovery. On this note, coinciding with impaired efferocytosis *in vitro*, Wong et al. observed greater neutrophil retention in the lungs and higher myeloperoxidase levels in the BALF of aged mice following influenza A virus infection (97). Interestingly, adoptive transfer of AMs from young mice into aged mice significantly reduced the degree of lung damage measured 3 days post influenza A virus challenge (97).

Associated with pathogen dissemination, impaired lung function and increased mortality (259, 260), down-regulation of ALOX5 (the gene responsible for the synthesis of the SPM lipoxin) and reduced production of the SPM protectin D1 (PD1) have been reported in murine models of severe influenza infection. Based in part on the fact that in these models administration of PD1 improved survival rates and pulmonary function (260), SPM treatment has been proposed as a therapy by which to promote the resolution of lung inflammation and reduce tissue damage in COVID-19 patients (261). Importantly, treatment regimens that include exogenous application of SPMs and inhibition of P38 MAPK have been shown in human and animal models to overcome the delay in inflammatory resolution that occurs as a consequence of aging and the presence of co-morbidities (225, 253, 256). Thus, it appears that resolution of inflammation can be manipulated in groups at high risk of severe COVID-19. However, as histological examination of lung tissue obtained from a SARS-CoV-1 infected patient found increased expression of plasminogen activator inhibitor-1 (262), a negative regulator of efferocytosis (263), there may be obstacles beyond impaired SPM generation that need to be overcome in order to successfully promote the resolution of coronavirus-induced inflammatory responses in older adults and those with inflammatory co-morbidities.

## Future Directions

### Immunesenescence and the Development of a COVID-19 Vaccine

A recent report by the World Health Organization provided information on the 26 candidate COVID-19 vaccines that are currently undergoing clinical testing and details of a further 139 that are in preclinical evaluation (264). The speed of COVID-19 vaccine research was highlighted by the fact that within 68 days of being declared a pandemic, results of the first animal and human based studies to test potential vaccines were published (265). Across rodents and non-human primates, the efficacy of an adenovirus-vectored vaccine encoding the spike protein of SARS-CoV-2 (266), a purified inactivated virus vaccine (267) and a series of DNA vaccines expressing different forms of the spike protein (268) have been tested, with preliminary results demonstrating the generation of robust humoral and cell-mediated responses that significantly reduce viral load and prevent the development of pneumonia (266, 267). Moderna Therapeutics recently announced the results of their phase 1 human trial of a potential COVID-19 vaccine (269). Using an mRNA vaccine that encodes for a pre-fusion stabilized form of the SARS-CoV-2 spike protein, the company reported seroconversion following a single dose in all 45 participants, with those who received two doses generating antibody levels akin to those measured in patients that have recovered from COVID-19 (269). However, it will be important to consider the impact of age and co-morbidities on the efficacy of any potential vaccine.

To date, a number of animal-based studies have investigated the effect of age on the efficacy of SARS-CoV-1 vaccines (270–272). In response to infection with homologous or heterologous viral strains, Bolles et al. found that aged mice vaccinated with an adjuvanted-double-inactivated whole SARS-CoV-1 virus were not completely protected against virus-induced mortality and exhibited both increased morbidity and pulmonary viral load when compared to young mice (270). Underlying this impairment in vaccine efficacy was a significant age-associated reduction in serum neutralizing antibody titres (270). However, in a related study, Sheahan et al. used a virus replicon particle vaccine platform that specifically targeted DCs, and showed that this strategy resulted in comparable antigen-specific IgG responses between young and aged mice and protected older mice from SARS-CoV-1-mediated clinical disease (272). Taken together, these data not only demonstrate the importance of testing any potential COVID-19 vaccine in all age groups but highlight how vaccine design will be critical for inducing protective antibody responses in aged hosts. On this note, a number of therapeutic strategies have been proposed and/or trialed in an attempt to combat the reduced efficacy of vaccinations against viral antigens in older adults (273). To date, these have included immunostimulant patches (274), the use of TLR agonists as adjuvants (275), the fusion of viral proteins with TLR agonists (276), high dose vaccination (277–279) and the use of PGD_2_ antagonists (242).

It is becoming increasingly recognized that obesity is a risk factor for infectious disease and poor vaccination responses (280–283). Data from mice (284–286) and human (287, 288) studies have reported reduced influenza vaccine efficacy in obese subjects, which in murine studies was associated with increased lung pathology, higher viral titres and greater mortality rates upon secondary infection (284, 285). Studies are underway to investigate methods of counteracting the negative effects of obesity on vaccine responses. Of note, whilst the use of adjuvants and/or high dose vaccination have been shown to increase neutralizing antibody titres in obese mice, the levels generated as well as the breadth and magnitude of the antibody response was significantly lower when compared to lean controls, ultimately resulting in reduced protection upon viral challenge (289). Thus, when viewed alongside the abovementioned age-related impairment in vaccine efficacy, these results imply that a “one size fits all” policy may not be appropriate for a COVID-19 vaccine, with high risk groups requiring a tailored vaccine designed to overcome the deficits of their remodeled immune systems.

### Enhancing Immune Function in Older Adults

Through pharmacological and non-pharmacological approaches, which include nutritional intervention (290, 291) and the administration of protein kinase inhibitors (204, 205, 225, 292, 293), clinical studies in older adults have shown it is possible to reverse immunesenescence.

Associated with reduced circulating frequencies of functionally exhausted PD-1 positive CD4^+^ and CD8^+^ T cells, Mannick et al. demonstrated a significantly enhanced serological response to influenza vaccination in older adults treated with the allosteric mammalian target of rapamycin (mTOR) inhibitor RAD001 prior to antigenic challenge (292). More recently, the same group reported that a combined therapy of RAD001 and BEZ235, a competitive mTOR inhibitor, significantly reduced the annualized rate of respiratory tract infections in adults aged ≥65 years (293). mRNA sequencing analysis of circulating leukocytes revealed this protective effect was accompanied by an up-regulation in genes related to anti-viral type I IFN signaling (293).

Oral administration of the potent and selective P38 MAPK inhibitor losmapimod has been shown to boost cutaneous immune responses in older adults. In a model of DTH, P38 inhibition was found to significantly increase VZV antigen specific immunity (205). Mechanistically, at the site of antigenic challenge, this improved immune response was associated with significantly reduced infiltration of PGE_2_ producing CCR2^+^ monocytes and increased T cell proliferation, whilst systemically, a significant decline in serum CRP levels was reported (204, 205). Importantly, losmapimod treatment also augments the resolution response of older adults (225), meaning that the improved immune response that would occur following P38 inhibition would not be offset by bystander tissue damage that would arise from the delay in the resolution of inflammatory responses that accompanies physiological aging (225).

Taken together, these studies demonstrate that it is possible to enhance anti-viral immunity and resolution responses in older adults. In the context of SARS-CoV-2 infection, these treatment regimens could be applied in a prophylactic manner to prevent the spread of COVID-19 and boost immune responses to future vaccines. Having already proven successful in elderly subjects, these therapeutic strategies have an advantage over many other potential treatments whose efficacy would be hampered by the remodeling of the immune system that occurs with age.

## Concluding Remarks

**Figure 1:**
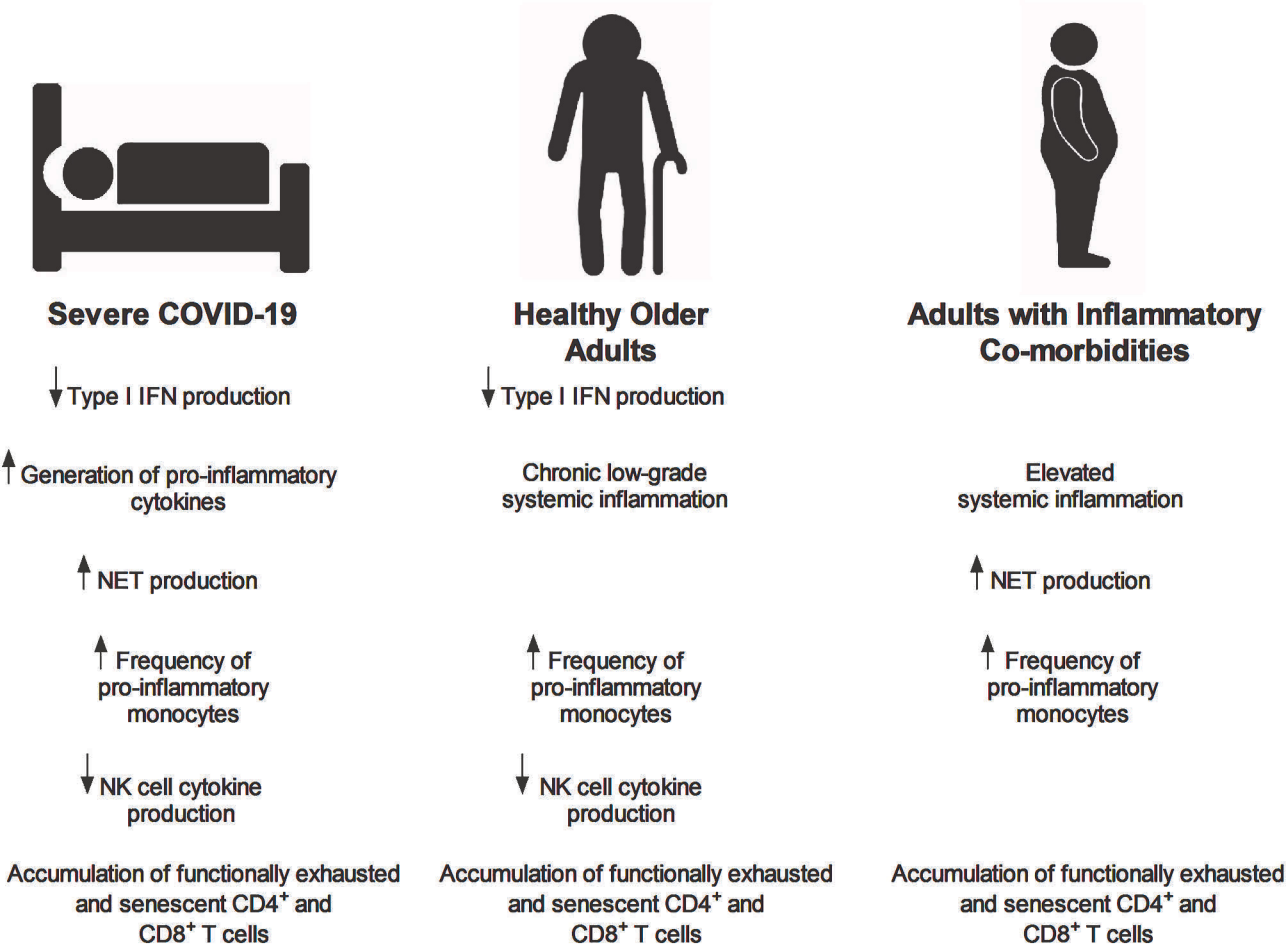
Immunesenescence: a risk factor for severe COVID-19? Similarities between the immune profile of patients with severe COVID-19, healthy older adults and adults with inflammatory co-morbidities (obesity and type 2 diabetes). IFN, Interferon; NET, Neutrophil extracellular traps; NK, Natural killer.

The similarities that exist between the immune response that precedes or accompanies the onset of severe COVID-19, and the re-modeled immune systems of older adults and those with inflammatory co-morbidities, lend support to the idea that immunesenescence may predispose to COVID-19 infection and disease severity (Figure 1). However, current evidence is at best circumstantial (202, 203), with the lack of cross-sectional and prospective studies examining the SARS-CoV-2-induced immune response in these high risk groups hindering our ability to address this hypothesis. That said, it appears that such studies are underway (10, 154, 294). For example, in a recent study, Liu et al. divided a cohort of 221 COVID-19 patients into two distinct age groups, and found older adults (≥60 years of age) presented with significantly elevated inflammatory indices (10). Furthermore, a study at University College London has acquired pre-infection blood and throat swab samples from people ≥70 years of age who will be assessed weekly for COVID-19 related symptoms (294, 295). Working on a hypothesis that prior exposure to coronaviruses may lead to an exaggerated immune response against SARS-CoV-2, one aim of the study is to determine pre-infection antibody titres against other coronaviruses (295). The results of this study, which also plans to search for biomarkers that are predictive of outcome in those subjects who develop COVID-19 (294, 295), will provide a much needed insight into how the immune system of older adults responds to SARS-Cov-2 and whether it is a contributory factor in patient outcome.

## Author Contributions

JH wrote the manuscript. JL critically appraised the manuscript. All authors have seen and approved the final submission.

## Conflict of Interest

The authors declare that the research was conducted in the absence of any commercial or financial relationships that could be construed as a potential conflict of interest.
